# Nutrition, Oral Health, and Chronic Diseases Inextricably Linked

**DOI:** 10.1093/geroni/igab046.967

**Published:** 2021-12-17

**Authors:** Teresa Marshall

**Affiliations:** University of Iowa, Iowa City, Iowa, United States

## Abstract

The 2020-25 Dietary Guidelines identified dental caries as a diet-related chronic disease of major importance. Preventing dental caries and other oral infectious diseases is critical to maintaining an individual’s capacity to chew food, consume nutrient-rich diets, and sustain optimal nutrition status. Pain and infection from dental caries complicates consumption of adequate amounts of fruits, vegetables, dairy, and lean protein recommended in the Dietary Guidelines. Nutrition and dietary intake can affect the development and integrity of oral cavity and progression of oral diseases. Increased snacking throughout the day in place of three-meals daily raises the risk of obesity and dental caries throughout the life cycle. Older adults who make routine oral health preventive practices, such as brushing, cleaning between teeth, drinking fluoridated water, and chewing sugarfree gum to increase saliva flow can reduce dental caries and oral infectious diseases. Professionals must also consider the impact of sugar-sweetened beverages and sugar intake.

